# Accurate Sex Identification of Ancient Elephant and Other Animal Remains Using Low-Coverage DNA Shotgun Sequencing Data

**DOI:** 10.1534/g3.119.400833

**Published:** 2020-02-27

**Authors:** Alida de Flamingh, Ashley Coutu, Alfred L. Roca, Ripan S. Malhi

**Affiliations:** * Program in Ecology, Evolution and Conservation Biology, University of Illinois at Urbana-Champaign (UIUC), Urbana IL, 61801,; † Department of Archaeology, University of Cape Town, 7700, RSA,; ‡ BioArCh, University of York, YO10 5NG, UK,; § Pitt Rivers Museum, University of Oxford, OX1 3PP, UK,; ** Department of Animal Sciences, UIUC, Urbana IL, 61801,; †† Carl R. Woese Institute for Genomic Biology, UIUC, Urbana IL, 61801,; ‡‡ Department of Anthropology, UIUC, Urbana IL, 61801

**Keywords:** XY karyotype, Rx ratio, *Loxodonta*, low-coverage, sex assessment, molecular sexing

## Abstract

Sex identification of ancient animal biological remains can benefit our understanding of historical population structure, demography and social behavior. Traditional methods for sex identification (*e.g.*, osteological and morphometric comparisons) may be ineffective when animal remains are not well preserved, when sex distinguishing characteristics have not yet developed, or where organisms do not exhibit sex-associated phenotypic dimorphisms. Here we adapt a method developed for human sex determination so that it can be used to identify the sex of ancient and modern animal taxa. The method identifies sex by calculating the ratio of DNA reads aligning to the *X* chromosome to DNA reads aligning to autosomes (termed the Rx ratio). We tested the accuracy of this method using low coverage genomes from 15 modern elephants (*Loxodonta africana*) for which sex was known. We then applied this method to ancient elephant ivory samples for which sex was unknown, and describe how this method can be further adapted to the genomes of other taxa. This method may be especially useful when only low-coverage genomic data are obtainable. Furthermore, because this method relies on only the *X* and not the *Y* chromosome, it can be used to determine the sex of organisms for which a reference genome was obtained from a female or for which only the *X* chromosome is reported. Such taxa include the domestic cat, sheep, goat, and horse; and non-domesticated animals such as the Sumatran orangutan, western lowland gorilla and meerkat.

Identifying the sex of animals can yield insights into population structure (Schoener and Schoener 1980; Bodkin *et al.* 2000), demographic histories (Heyer *et al.* 2012) and social interactions (Pedersen *et al.* 1990; Lonsdorf *et al.* 2014). It can add to knowledge of extinct and extant animal populations and reveal how they have changed across time. Sex identification can aid our understanding of extinct animal biology (Allentoft *et al.* 2010), past hunting practices and domestication (Collier and White 1976; D’Errico and Vanhaeren 2002). For many ancient or historical samples, however, the sex of specimens is unknown. Sex identification may be hindered when remains are very degraded or only partially preserved, when remains are from young individuals where sex distinguishing characteristics have yet to develop, or when remains are from taxa that do not exhibit phenotypic sexual dimorphism (Hamilton *et al.* 1986). Such factors may preclude sex identification through traditional methods such as osteological or morphometric comparison (measurements of skeletal ratios/aspects) (Safont *et al.* 2000; Rogers 2005; Bruzek and Murail 2006). Molecular sex identification circumvents these issues, requiring only a small sample for DNA analysis. For ancient samples with a low quantity and quality of DNA (Quincey *et al.* 2013), molecular methods test for DNA authenticity by determining whether amplified DNA exhibits damage patterns typical of ancient DNA (Jónsson *et al.* 2013). Molecular methods therefore permit sex identification of degraded or partial specimens, from young and from sexually monomorphic taxa. Molecular sex identification methods have involved the analysis of genes associated with male and female sex chromosomes in birds (Griffiths *et al.* 1998; Fridolfsson and Ellegren 1999), reptiles (Quinn *et al.* 2009), mammals (Sullivan *et al.* 1993; Gibbon *et al.* 2009), and fish (Chen *et al.* 2007). For example, in some mammals, molecular sex identification involves differentiating between amelogenin gametologues on the *X* and *Y* chromosomes (Sullivan *et al.* 1993; Gibbon *et al.* 2009).

For sex identification of archeological human remains, Mittnik *et al.* (2016) developed a method that uses low coverage whole genome data to calculate the Rx ratio, which compares DNA sequence reads that align to the *X* chromosome to DNA sequence reads that align to autosomal chromosomes. The Rx ratio is different for females and males, since they have two or one *X*-chromosomes, respectively. The Rx ratio would be expected to be ca. 1.0 for females and 0.5 for males. Mittnik *et al.* (2016) identified individuals as female if the Rx 95% CI lower bound was higher than 0.80, and as male if the 95% confidence interval (CI) upper bound for Rx was lower than 0.60.

Here, we present an extension and expansion of the method of Mittnik *et al.* (2016) to permit sex identification of ancient and modern samples of non-human taxa. We adjust the Rx equation to mathematically account for different chromosome numbers across animal taxa. Our method, in principle, allows for accurate sex determination of any organism with *XY* sex determination for which a reference genome is available with chromosome-level resolution. We verify the method using low-coverage genomes from 15 modern elephants for which sex is known, and apply this method successfully to low-coverage genomes of ten ancient elephant ivory samples for which sex was unknown. These ancient ivory samples are from a 16^th^ Century shipwreck uncovered in Namibia and believed to be the *Bom Jesus*, a Portuguese trading ship lost in 1533 en route to India (Werz 2010; Alves 2011).

## Materials and Methods

### DNA extraction and shotgun sequencing

DNA was extracted from skin biopsy samples from 15 African elephants for which sex was recorded in the field when the samples were collected. The modern elephant samples were from nine females and six males (Table 1). Genomic libraries were constructed for the 15 modern elephants at the UIUC Core Sequencing Facility using TruSeq DNA library preparation. To generate low-coverage genomes for the modern elephant samples, we sequenced the 15 samples as part of a larger pool of samples in a single HiSeq 4000 lane (150bp paired-end). DNA from the ancient ivory was extracted following methods described in Cui *et al.* (2013). Cui *et al.* (2013) provide details, for example, on starting template amounts (0.20g per ancient sample) and treatment protocols. Ancient DNA work (extractions and genomic library preparation) was conducted in the Malhi Ancient DNA Laboratory, which is dedicated exclusively to studies involving ancient DNA, at the Carl R. Woese Institute for Genomic Biology, University of Illinois at Urbana-Champaign (UIUC). All rounds of DNA extraction included a negative control to verify that reagents and equipment were not contaminated and that there was no cross-contamination between samples, and not more than eight samples were processed at any one time. Libraries for the ten ancient ivory samples were constructed using the NEBNext Ultra II DNA Library Prep kit and NEBNext Multiplex Oligos (Unique Dual Indexes) for Illumina. Because ancient DNA are prone to have cytosine to uracil nucleotide base changes (Hofreiter *et al.* 2001), the extracted ancient DNA was pre-treated with USER (Uracil-Specific Excision Reagent) enzyme. The modern and ancient libraries were pooled separately, and each pool was shotgun sequenced on a HiSeq 4000 platform at the UIUC Core Sequencing Facility.

**Table 1:** Known sex of modern elephants, and predicted sex using the Rx ratio

Sample ID	Rx ratio*^a^*	95% CI*^b^*	Known sex	Predicted sex
DS1531	0.9348111	0.9241519	Female	Female
		0.9454702		
DS1548	0.4844121	0.4781392	Male	Male
		0.4906849		
DS1514	0.9300965	0.9194486	Female	Female
		0.9407445		
DS1543	0.4918646	0.4866054	Male	Male
		0.4971237		
DS1506	0.4878141	0.4826157	Male	Male
		0.4930124		
LO3503	0.9321284	0.9215853	Female	Female
		0.9426715		
LO3509	0.9415045	0.9308473	Female	Female
		0.9521617		
LO3511	0.9287396	0.917744	Female	Female
		0.9397352		
LO3521	0.8712463	0.8574852	Female	Female
		0.8850075		

a The Rx ratio compares DNA sequence reads that align to the *X* chromosome to DNA sequence reads that align to autosomal chromosomes, and would be expected to be ca. 1.0 for females and 0.5 for males.

b The top value represents lower bound of the 95% Confidence Intervals (CI) and the lower value represents the upper bound of the 95% CI.

### Bioinformatic analyses and Rx based sex identification

Sample reads were de-multiplexed and trimmed using the program FastP v.0.19.6 (Chen *et al.* 2018) to have a minimum sequence length of 25bp. Reads were aligned to the chromosome-level assembly of the African savanna elephant genome (*Loxodonta africana* assembly Loxafr4.0, Broad Institute (Palkopoulou *et al.* 2018)) using bowtie2 (Langmead and Salzberg 2012) with the local alignment option, and capping fragment length at 1000bp. Aligned sequences were transformed to BAM format in SAMtools v. 1.1 (Li *et al.* 2009). Using SAMtools, BAM files were filtered to remove unmapped reads and reads with a quality score less than 30, then sorted and indexed, with PCR duplicates marked and removed with the Picard Toolkit v. 2.10.1. Index statistics for BAM files were generated using “idxstats” in SAMtools (Li *et al.* 2009).

**Figure 1:**
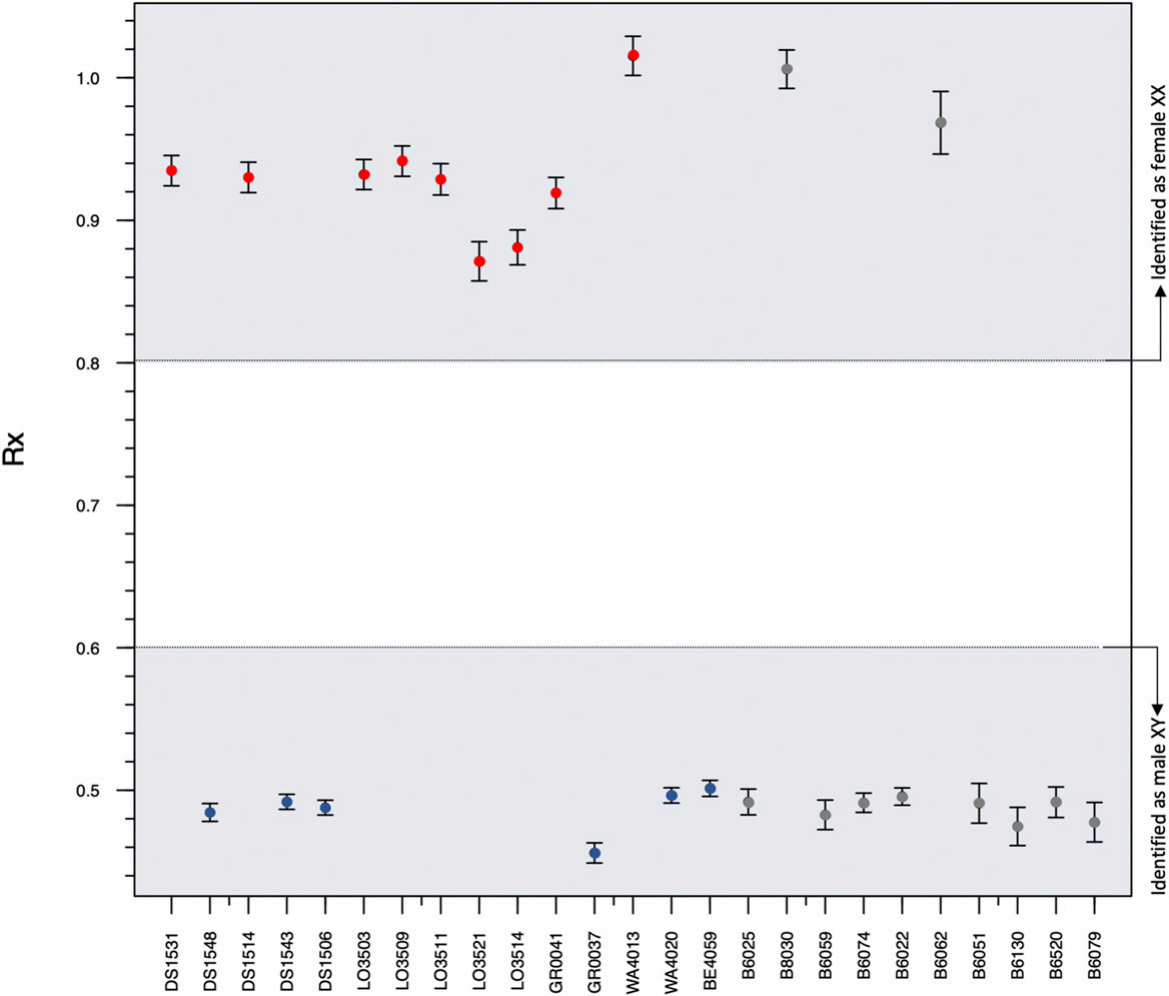
Rx values for modern elephants of known sex (red and blue) and ancient elephant samples of unknown sex (gray). Rx is the ratio of sequence read alignments to the *X* chromosome compared to sequence read alignments to all autosomes. Rx values are shown for low coverage modern elephant genomes of known males (blue) and females (red), and for ancient elephant genomes for ivory samples of previously unknown sex (gray). An Rx ratio with an upper 95% CI of less than 0.6 indicates male sex, and an Rx ratio with a lower 95% CI that is greater than 0.8 indicates female sex.

The Rx_identifier.r script of Mittnik *et al.* (2016) was modified to accommodate the number of chromosome pairs found in elephants, which is different from the number in humans, for which the script was originally developed (see Supplementary Appendix 1 for a stepwise protocol of how to modify this script for any organism that has a chromosome-level reference genome and *XY* sex determinism). We verified that the row numbers in the Rx_identifier script corresponded to the correct chromosome identities in our sorted idxstat files. The modified Rx_identifier.r script was then implemented using the program R v. 3.3.3 (R-Development-Core-Team 2017) and the indxstat files as input. Output statistics for each sample included the Rx ratio, and sex identification based on the data ranges of Mittnik *et al.* (2016), where a sample was identified as male if its 95% confidence interval (CI) upper bound for Rx was lower than 0.60 and identified as female if its Rx 95% CI lower bound was higher than 0.80. The 95% CI was computed as Rx± 1.96 SE (standard error), where the SE measures the amount of variability in the Rx mean compared to autosomes (22 for humans, 27 for elephants). We determined whether sequence coverage was sufficient by performing a linear regression of the number of sequenced and mapped reads on each chromosome against the number of reference reads. Output statistics were visualized by plotting individual Rx ratios (Figure 1) using R v. 3.3.3 (R-Development-Core-Team 2017). The bioinformatic analyses were repeated using BWA (Li and Durbin 2010) to check for inconsistencies that could be associated with sequence aligner choice, but no inconsistencies were observed and sex identification was completely consistent between the two analyses. Ancient DNA damage patterns were verified by aligning trimmed reads to the African savannah elephant genome (LoxAfr 4.0) using BWA (Li and Durbin 2010) and quantifying damage in mapDamage2 (Jónsson *et al.* 2013) using a fragment size of 70bp.

To determine how effective the Rx method is for determining the sex of samples with even lower coverages that ours, we subsampled the existing ancient ivory data to include datasets of approximately 10 000 and 1 000 reads. We used Sambamba (Tarasov *et al.* 2015) to subsample datasets, and reanalyzed the subsampled datasets using the Rx method.

### Data availability

All idxstats files for modern and ancient elephant genomes, and all Rx ratio result files for the 10 000- and 1000-read subsampled files are available from GSA Journals figshare portal. The most recent update of the savanna elephant reference genome (LoxAfr4) is available at ftp://ftp.broadinstitute.org/pub/assemblies/mammals/elephant/loxAfr4/. R-scripts and a step-by-step description of how to adapt the R-script to any species chromosome-level genome can be found at https://github.com/adeflamingh/de_Flamingh_et_al_2020_G3.git or as Appendix 1 and 2, or as part of the supplementary material on GSA Journals figshare. The study was conducted under the University of Illinois Institutional Animal Care and Use Committee approved protocol number 18042. Samples were imported through a CITES permit. Supplemental material available at figshare: https://doi.org/10.25387/g3.11837157.

## Results

Although all ancient samples were pre-treated with USER enzyme, which may potentially mask damage patterns (see Methods), DNA damage patterns in the ancient ivory were still evident and typical of ancient DNA (Supplementary Figure 1). Each of the ancient samples showed increased rates of C to T and G to A mismatches relative to the reference genome, as would be expected in authentic ancient DNA.

The adapted Rx_identifier.R script (Supplementary Appendix 2) was able to identify sex for all 15 modern individuals (nine females, six males) with 100% accuracy (Figure 1; Table 1). For the ancient DNA remains, the adapted script identified eight individuals as male, and two individuals as female (Figure 1; Table 2). Linear regressions of the number of reference genome reads with the number of mapped reads resulted in significant F-statistic values (*P* < 0.001) for both modern and ancient remains, indicating that the sequence coverage for all genomes was sufficient for accurate sex determination.

**Table 2:** Predicted sex of ancient elephant samples using the Rx ratio

Ancient sample ID	Read count	Rx ratio*^a^*	95% CI*^b^*	Predicted sex
B6025a	991,719	0.4917869	0.4827309	Male
			0.5008429	
B8030	6,186,805	1.00599	0.9925127	Female
			1.019467	
B6059	3,672,375	0.4827319	0.4723486	Male
			0.4931151	
B6074	864,649	0.4912222	0.4844642	Male
			0.4979802	
B6022	1,848,409	0.4955726	0.4894958	Male
			0.5016494	
B6062	919,574	0.9684064	0.9464218	Female
			0.9903909	
B6051	460,668	0.4908337	0.476899	Male
			0.5047683	
B6130	17,797,452	0.4745888	0.4611754	Male
			0.4880023	
B6520	141,855	0.4915878	0.4808738	Male
			0.5023018	
BB6079	5,729,007	0.477581	0.4637086	Male
			0.4914535	

a The Rx ratio compares DNA sequence reads that align to the *X* chromosome to DNA sequence reads that align to autosomal chromosomes, and would be expected to be ca. 1.0 for females and 0.5 for males.

b The top value represents lower bound of the 95% Confidence Intervals (CI) and the lower value represents the upper bound of the 95% CI.

The Rx ratio method effectively identified the sex when using data files with > 100 000 reads (all 95% CI are within the specified Rx cut-off values; Supplementary figure 2). The method was mostly effective when using subsampled ancient ivory data files with 10 000 reads (only sample B6079 had 95% CI outside of Rx cut-off values), but proved less effective when using subsampled datasets with 1000 reads (the span of the 95% CI increased for all samples and four samples had 95% CI outside of Rx cut-off values; Supplementary figure 2).

## Discussion

We adapted a method previously developed Mittnik *et al.* (2016) for sex identification of human remains for use with non-human taxa, and successfully identified the sex of modern and ancient elephants from low coverage genome data. Because the Rx ratio sex identification method presented in this study relies only on the *X* and not the *Y* chromosome, it can be used to identify the sex of organisms in which the reference genome was obtained from a female animal or where only the *X* and not the *Y* chromosome is reported in the reference genome assembly for the taxon. Such taxa would include (but not be limited to) the domestic cat, sheep, goat, horse, dromedary camel, European rabbit; and also include many wild animals such as the Sumatran orangutan, western lowland gorilla, gelada and meerkat (Supplementary Table 1). Being able to identify the sex of samples could benefit agricultural studies on domesticated animals, and could inform conservation initiatives that focus on non-domestic wildlife. Because this method is amenable to low coverage data from low quantity DNA (*e.g.*, ancient or degraded DNA), it can be employed as a non-invasive approach to identifying sex of endangered or rare species, for example, through the analysis of DNA from hair tufts (McKelvey *et al.* 2006; Stanton *et al.* 2016) or herbivore scat (Huber *et al.* 2002). By requiring only minute quantities of DNA as a starting template, the method could be extended to other types of degraded DNA such as archival samples from museum collections (Wandeler *et al.* 2003; Bi *et al.* 2013) or forensic samples (Jobling and Gill 2004; Alaeddini *et al.* 2010).

Sex identification using the Rx ratio could be adapted to any taxa that exhibit *XY* sex determination for which a chromosome-level genome assembly is available. It should be possible to further extend the method to taxa that have a *ZW* sex determination system, in which males are the homogametic sex *ZZ*, and females have *Z* and *W* chromosomes. Such taxa include birds (Chue and Smith 2011), amphibians (Nakamura 2009) and crustaceans (Cui *et al.* 2015). For *ZW* sex determination systems, individuals should be identified as male (*ZZ*) if the lower bound of their 95% Rx ratio CI is approximately 0.8 or higher, and female (*ZW*) if the upper bound of the 95% Rx ratio CI is approximately 0.6 or less. For *ZW* sex determination the script should be adapted so that the *Z* chromosome replaces the *X* chromosome in the Rx_identifier.R script, and the *W* chromosome replaces the *Y* chromosome (if it is present in the reference genome). Again, since the script could rely only on the *Z* chromosome and not the *W* chromosome, this method may be used on any individual, male or female, with *ZW* sex determination if there is a chromosome-level reference genome assembly available for that species. Future studies would be needed to validate the use of the adapted script on animals other than elephants or humans.

We investigated whether the Rx method can effectively identify the sex of individuals when using genome coverage even lower than that of the ancient ivory samples. We found that there is a substantial broadening of the 95% CI as the read count of the data file decreases (Supplementary figure 2). We suggest that the Rx cut-off values presented by Mittnik *et al.* (2016), and in this paper, may be useful indicators of the ability of Rx script to accurately and precisely identify individual sex, and caution users to be less confident in sex identification if the confidence intervals extend beyond these cut-off values.

The Rx ratio method was successfully used here on low coverage genomic data from both modern and ancient (Supplementary Table 2) elephants. The ability to accurately identify sex based on low coverage data may be especially useful with ancient samples with DNA of low quantity and quality (Quincey *et al.* 2013), and for studies that index and pool a large number of individuals for sequencing (*e.g.*, PoolSeq studies). Such studies may have low coverage per individual, but many individuals may be indexed and pooled to represent a population. The limited requisites and ease of adaptation and implementation of this method would allow for convenient and effective identification of the sex of modern and ancient animal remains.
